# Planarian nociception: Lessons from a scrunching flatworm

**DOI:** 10.3389/fnmol.2022.935918

**Published:** 2022-07-26

**Authors:** Guillaume Reho, Vincent Lelièvre, Hervé Cadiou

**Affiliations:** Institut des Neurosciences Cellulaires et Intégratives, UPR 3212, Centre National de la Recherche Scientifique, Strasbourg, France

**Keywords:** planarians, nociception, drug screening, animal model, opioids, TRP ion channels, behavior, invertebrate

## Abstract

In addition to being studied for their exceptional regeneration abilities, planarians (i.e., flatworms) have also been extensively used in the context of pharmacological experiments during the past century. Many researchers used planarians as a model system for the study of drug abuse because they display high similarities with the nervous system of vertebrates at cellular and molecular levels (e.g., neuronal morphology, neurotransmitter ligands, and receptor function). This research field recently led to the discovery of causal relationships between the expression of Transient Receptor Potential ion channels in planarians and their behavioral responses to noxious stimuli such as heat, cold or pharmacological analogs such as TRP agonists, among others. It has also been shown that some antinociceptive drugs modulate these behaviors. However, among the few authors that tried to implement a full behavior analysis, none reached a consensual use of the terms used to describe planarian gaits yet, nor did they establish a comprehensive description of a potential planarian nociceptive system. The aim of this review is therefore to aggregate the ancient and the most recent evidence for a true nociceptive behavior in planarians. It also highlights the convenience and relevance of this invertebrate model for nociceptive tests and suggests further lines of research. In regards to past pharmacological studies, this review finally discusses the opportunities given by the model to extensively screen for novel antinociceptive drugs.

## Introduction to planarians

**FIGURE 1:**
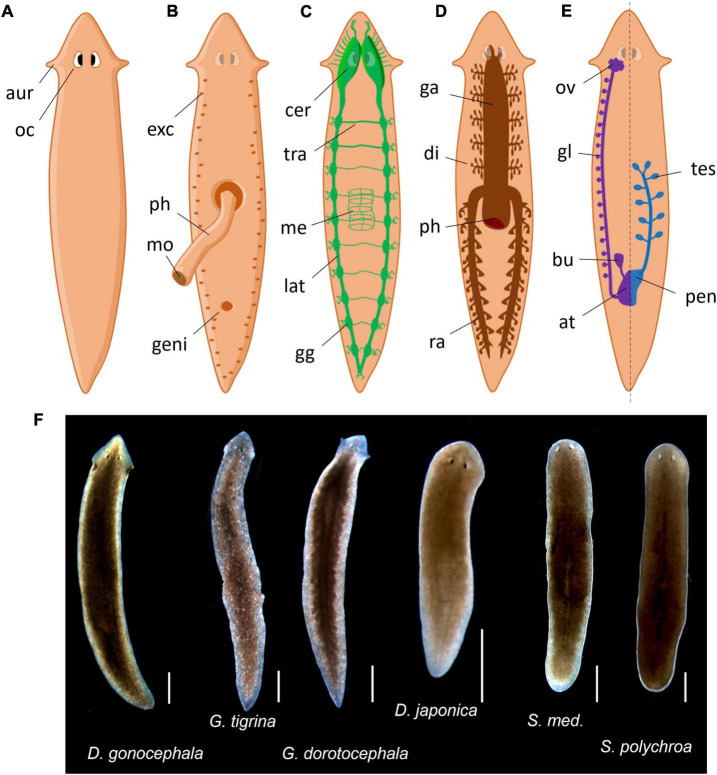
Common planarian representations. **(A)** Dorsal exterior view. aur, auricles; oc, ocelli. **(B)** Ventral exterior view with extruded pharynx. exc, excretory pores; ph, pharynx; mo, mouth; geni, genital pore. **(C)** Nervous system. cer, cerebral ganglions; tra, transversal nerve; me, pharyngal mesh of neurons; lat, lateral nerve cord; gg, lateral ganglions. **(D)** Gastrovascular system. ga, gastrovascular cavity; di, diverticulum; ph, pharynx; ra, posterior ramus. **(E)** Reproductive system, female is represented on the left and male on the right but both are present symmetrically. ov, ovary; gl, yolk glands; bu, bursa copulatrix; at, genital atrium; tes, testis; pen, penis. **(F)** Photos of common planaria species from our laboratory (scale: 1 mm).

The term *planaria* (or planarians) encompasses flatworms (platyhelminthes phylum) within the *Tricladida* order. The other three out of the four major classes of flatworms – *Trematoda*, *Cestoda*, and *Monogenea* – contain mostly parasitic species but *Tricladida* are free-living organisms. They used to be classified as *Turbellaria* due to their gliding movement using ventral cilia but this class is now considered paraphyletic (de Vries and Sluys, 1991). Triclads include marine (*Maricola*), land (*Geoplanidae*) and freshwater flatworms. Most of them might be called planarians (e.g., “land planarians” for *Geoplanidae* species), but the term *planaria* or planarians in research models mostly refers to freshwater species, especially from the Dugesiidae family. Common species used as model animals come from the three Dugesiidae genus *Girardia*, *Dugesia*, and *Schmidtea*, such as *Girardia tigrina*, *Girardia dorotocephala*, *Dugesia japonica*, or *Schmidtea mediterranea* (Figure 1F). Note that *tigrina* and *dorotocephala* are still sometimes referred as from the “*Dugesia*” or “*Dugesia (Girardia)”* genus by common practice, but *Girardia* is not classified as a subgenus of *Dugesia* anymore (de Vries and Sluys, 1991).

As they do not have a body cavity, planarians are considered as acoelomates. Their digestive system consists of a three-branched gastrovascular cavity, hence the term triclad (Figure 1D). It is shaped as an inverted Y that opens in the center of their ventral side with a muscular appendage called the pharynx. The pharynx is dedicated to food intake, to initiate digestion and finally to expel the leftovers. This structure possesses a dedicated mesh of neurons that guide the animal for food-localization movements (Miyamoto et al., 2020). After crossing the pharynx, food is then distributed throughout the gastrovascular cavity to provide nutrients by diffusion during the digestion. Outside lab conditions, they mostly feed on other small invertebrates (some are even cannibalistic) and debris. Regarding their reproductive behavior, all planarians are true hermaphrodites (Figure 1E). Despite having functional male and female reproductive systems, they can usually switch between sexual and asexual reproduction but some others only reproduce asexually (Ramm, 2017). Asexual reproduction is usually done by fragmentation or fissioning of the animal, resulting in further regeneration of missing parts. Planarians mostly perform fissioning when the population is decreasing and cease fissioning when the population is crowded (Best et al., 1969). They also have a basic excretory system composed of flame cells and mucus-producing cells called rhabdites – hence their classification in the Rhabditophora class (Figure 1B). This system is not only crucial for locomotion (Sugimoto, 2010) but may also participates to defense mechanisms against infections or predators as in other species (Cochet-Escartin et al., 2015).

Their nervous system consists of a “brain” located in the anterior part of the animal from which originates two ventral longitudinal nerve cords running on the entire length of the animal (Figure 1C; Agata, 2008). These nerve cords also branch out in transversal nerves, giving their central nervous system the appearance of a grid or a ladder. The so-called brain is characterized by two cerebral ganglions linked by a cerebral commissure. The term “brain” in *planaria* is often used as a shortcut for cerebral ganglions, even though there are arguments for actually using the term “brain” as an accurate depiction of its structure (Sarnat and Netsky, 2002). Sensory neurons branch out from the nerve cords all along the body. The brain also extends nine branches out of each lobe to the head margin that primarily serve as sensory neurons, especially as chemical and mechanical sensors, present in the “auricles” in some species (ears-like structure particularly visible in *G. dorotocephala*). Above the brain extend the eye spots, actually called ocelli (Figure 1A), which typically look cross-eyed because of their two cell type compositions: photoreceptors (white cupola) and pigmented cells (dark spots) (Saló et al., 2002; Inoue et al., 2004). Planarians are mostly nocturnal animals, hence showing more activity in the dark period (Lombardo et al., 2011; Hinrichsen et al., 2019). These photoreceptors sense photoperiodic information but also allow typical defensive photophobic reactions to light (Paskin et al., 2014). However, planarians also show behavioral responses mediated by extraocular photoreception, especially to UV light (Birkholz and Beane, 2017).

One of the key features of this model is that planarians display huge regenerative capacities. They possess populations of pluripotent stem cells called *neoblasts* that can be triggered by lesions and form a regenerative area called *blastema* (Reddien and Alvarado, 2004; Reddien, 2018). They are able to regenerate every tissue, thus allowing the complete regeneration of the animal’s missing part, to the extent of complete nervous system regeneration and even neural networks reconstruction (Cebrià, 2007; Nishimura et al., 2007). Even small parts from almost any tissue can regrow into a complete individual from limited number of cells and signaling gradients (Reddien and Alvarado, 2004; Ivankovic et al., 2019). From a comparative neurobiology point of view, it is quite striking that morphogenetic signals initiating planarian regeneration are very similar to the intrinsic factors shaping up the human brain during development (Currie et al., 2016; Reddien, 2018).

## Planarian research throughout centuries

Planarians have been a useful model in numerous domains of biology for the past three centuries. Their first known mentions date back to the late 18th century (Müller, 1776; Pagán, 2014). The phenomenon that brought researchers’ attention was their extraordinary regeneration abilities. In the early 19th century, Dalyell (1814) already wrote the now famous citation “It may almost be called immortal under the edge of the knife.” Later, at the turn of the 20th century, multiple scientists were publishing extensive descriptions of the worm’s morphology, physiology and behavior (Bardeen, 1901; Pearl, 1903). By the middle of the century, research on planarians ranged as far as tropism (Viaud, 1948), magnetobiology (Brown, 1962), conditioning and learning (Corning and Kelly, 1973), phylogenetics (Minelli, 1977), and behavior (Ogren, 1956), as only a few non-exhaustive examples. Regeneration studies also continued to thrive from planarians, elucidating the cellular nature of regeneration and the fundamental properties of stem cells (Elliott and Alvarado, 2018). In the meantime, their use in pharmacology and toxicology studies has increased steadily (Pagán, 2017). Indeed, planarians’ cerebral ganglions can actually constitute a brain analog (Sarnat and Netsky, 2002). An idea popularized by Pagán (2014) in “The First Brain,” where the author described flatworms as the “simplest” animals with a central nervous system. Yet, they share a lot of common characteristics with vertebrates, such as multipolar neurons with a unique axon, extensive dendritic branching with developed dendritic spines, a majority of chemical synapses and a low-frequency spontaneous electrical activity (Sarnat and Netsky, 1985, 2002). These characteristics are not shared with most higher-grade invertebrates. Besides, they possess many of the neurotransmitters that are present in vertebrates (Ribeiro et al., 2006; Buttarelli et al., 2008), as well as similar interactions between their pathways (Carolei et al., 1975). They also serve as a great model for a wide range of drugs and for poly-drug testing, as they allow the study of multiple compounds at once and with higher accuracy of pharmacokinetic factors (absorption, distribution, metabolism, excretion; i.e., ADME) than with higher models like rodents or primates (Raffa, 2008). Hence, *planaria* has been a model animal for hundreds of pharmacological studies with a particular focus on substances of abuse like cocaine, amphetamines or nicotine, as they display signs of withdrawal and tolerance in the same way more evolved animals do (Pagán, 2017).

Planarians glide on surfaces using ciliated cells located on their ventral side, on a layer of mucus that they constantly produce (Rompolas et al., 2010; Sugimoto, 2010). It has been long known that when exposed to a harmful stimulus (either mechanical, chemical, or electrical), planarians display clear behavior changes (Pearl, 1903). When disrupted, the animals will usually produce more mucus, stop gliding smoothly and adopt various body shapes, movements, and postures. These reactions highly resemble nocifensive behavior seen in other invertebrates or even vertebrates, including mammals (Kavaliers, 1988). Yet, planarians are still not considered as animal models for nociception testing, nor clearly presented as having a nociceptive system or pathway. In the next sections, we will discuss why there are many reasons to consider that planarians may actually present a nociceptive system and that many experiments can be done to decipher more precisely its mechanisms, once again supporting the usefulness and the ethical relevance of this invertebrate model in the nociception and pain research domain.

## Nociception in vertebrates vs. invertebrates

The vast majority of nociception and pain research has been and is done in mammals, and rodents in particular (Mogil, 2009). In this context, nociceptors represent the free nerve endings that specialize in the detection of noxious stimuli and forward this information to the spinal cord before being sent to higher cortical regions. Thus, nociception is defined as “the neural process of encoding noxious stimuli” (Terminology | International Association for the Study of Pain [IASP], 2022). Nociceptors are usually defined by the composition of their axon, either myelinated (fast-conducting “Aδ” fibers) or unmyelinated (slower-conducting “C” fibers) and express multiple ion channels specialized in detecting noxious compounds or stimuli. These ion channel receptors include mainly the transient receptor potential (TRP) ion channels among others, such as ASICs or P2X channels (Tracey, 2017). Pain, on the other hand, is defined as “an unpleasant sensory and emotional experience associated with, or resembling that associated with, actual or potential tissue damage” (Terminology | International Association for the Study of Pain [IASP], 2022). Thus, as an over-simplification, pain is usually the result of sufficient nociceptive input that is integrated in the cortex as emotionally unpleasant. This definition is particularly relevant for humans that communicate but lacks inclusivity toward animal pain as it involves conscious representations that we cannot yet interpret from animal behavior. The animal pain research field tries to decipher whether animals experience pain in similar ways as humans do through lists of criteria, and even though so-called pain testing paradigms exist (e.g., place preference test or analgesia self-administration), they cannot conclude on certainty that they experience pain the same way humans do (Sneddon et al., 2014). Conclusions mostly revolves around ethical considerations and humane use of animals in research as a precautionary principle. This should also be valid toward the invertebrates’ pain and nociception research field. Indeed, while vertebrates’ and especially mammalians’ pain is mostly accepted as a highly probable concept, invertebrates’ nociception is unfortunately way less regarded (Elwood, 2019).

Looking back at nociceptors, invertebrates do not possess such fibers classification and their sensory neurons might not be as specialized. Nonetheless, some of their neurons also express nociceptive ion channels. Indeed, TRP ion channels were initially discovered in *Drosophila* photoreceptors (Cosens and Manning, 1969; Katz et al., 2017). They are highly conserved along the animal kingdom and seem to be at the base of many noxious sensing systems (Peng et al., 2015; Arenas et al., 2017). Moreover, the strict definition of nociception previously discussed does not apply exclusively to vertebrates. Many animals, including very low species on the phylogenetic tree of life, show signs of what seems to be defense mechanisms when exposed to unusual environmental conditions or predators (Kavaliers, 1988). Many other “pain perception” criteria have been experimentally tested on multiple invertebrates models, such as protective, avoidance or trade-off behaviors (Sneddon et al., 2014; Elwood, 2019). However, only a few invertebrate models actually have a complete or even partial description of their nociceptive system, usually involving more or less specialized neurons (Burrell, 2017; Tracey, 2017).

In one of the most well studied invertebrate model *Drosophila*, sensory neurons are classified as class I to IV based on their dendritic arborescence. The class IV neurons are particularly involved in noxious heat, mechanical and chemical detection and project to the nerve cords of the CNS (Im and Galko, 2012). In the nematode *Caenorhabditis elegans*, nociception is based on behavioral responses following aversive cues. It involves the polymodal ASH and the mechanosensory PVD neurons (Tobin and Bargmann, 2004). In the leech *Hirudo verbana*, sensory neurons are classified as N cells (Nociceptive), T cells (Touch), and P cells (Pressure) based on their threshold of activation following mechanical stimuli (Blackshaw et al., 1982). In the mollusk *Aplysia*, its so-called LE sensory neurons are particularly involved in noxious mechanical stimuli detection (Illich and Walters, 1997). In the squid *Loligo*, mechanosensitive nociceptors are located in its fins (Crook et al., 2013). Other models have been studied, mostly less extensively, and can be used to compare the phylogeny of nociceptors, such as other mollusks, fish, amphibians, or reptilians. These studies include different sets of tests, sometimes only partially carried out, such as behavioral responses to noxious stimuli, electrophysiological responses of sensory afferent fibers in teased nerve preparations or whole cell patch clamp of sensory neurons (Smith and Lewin, 2009).

These specialized neurons in each species have been identified as nociceptive because they display all, or a part of, characteristics similar to those of vertebrates: an implication in behavioral noxious responses, a specific expression of TRP-like ion channels or other nociceptive-related receptors, a high threshold to induce an action potential, a slow adaptation, a specific activation following a noxious stimuli and a potential modulation. Indeed, while nociceptors usually display specific physiological characteristics, they also display high plasticity. For example, a common modulation process is sensitization, which lowers the activation threshold and/or augments the action potential frequency, induced by continuous stimuli, inflammatory soup (e.g., ATP, cytokines, acidic compounds, etc.) or phosphorylation of TRPs’ intracellular segments (Smith and Lewin, 2009).

## Evidence for nociception in planarians

Based on previous observations about invertebrate models of nociception, we suggest here that planarians show sufficient arguments for the model to be considered for nociceptive tests.

### Behavior

First and foremost, planarians seem to display a variety of reproductible behaviors in response to noxious stimuli. As planarians do not have movable ocelli or facial expressions, almost everything we know about planarian behavior comes from their locomotion. Therefore, we can only observe the way they move or the body shape they take. Planarians also display negative phototaxis, which allows for locomotion inducing and tests such as place preference or place conditioning experiments (Paskin et al., 2014; Dziedowiec et al., 2018; Zewde et al., 2018). Further description of planarian behavior studies will be discussed in section “Planarian behavioral metrics.”

### Transient receptor potential ion channels

#### The presence of transient receptor potential ion channels in planarians

Secondly, the TRP ion channels present in many sensory and nociceptive pathways are highly conserved among the animal kingdom so they were the main target to identify a possible nociceptive pathway in planarians. TRPs are membrane ion channels that have a non-selective permeability to cations and represent the main ion channel family responsible for the detection of noxious stimuli on nociceptors (González-Ramírez et al., 2017). They form as tetramers and are regrouped into different families based on their amino acid sequence and structure. The main TRP families involved in nociception are TRPA (ankyrin), TRPV (vanilloid), and TRPM (melastatin). The presence of TRP channels in planarians were first assumed from the reactions of the animals to TRP agonists (Rawls et al., 2007a), but the expression of planarian TRP family genes have since then been observed throughout the whole body of the animals by *in situ* hybridization (Inoue et al., 2014). Notably, TRPAa and TRPMa displayed a scattered expression on the periphery and a denser expression in the head suggesting their particular involvement in sensory neurons, while TRPVa pattern of expression is more homogenously distributed throughout the body in mesenchymal cells (Inoue et al., 2014).

On the other hand, in planarians, protein knockdown can be achieved by interference RNA technology (RNAi) using double-stranded (ds) RNA microinjected directly in the host tissue (Sánchez Alvarado and Newmark, 1999) or, in a broader way, using food infected with dsRNA-producing bacteria (Newmark et al., 2003) or supplemented with *in vitro* synthetized dsRNA (Rouhana et al., 2013). After injection or ingestion, RNA segments will diffuse throughout the animal and hybridize with the target protein’s transcript, effectively blocking its translation and thus the formation of the protein of interest. Hence, multiple gene expression marking and repressing of TRP channels have been done in planarians thanks to the continuous efforts in the sequencing of their genome and its availability (Rozanski et al., 2019).

#### The role of transient receptor potential ion channels in planarians

Performing such knockdown on planarians’ TRPA1 channels, commonly involved in noxious heat sensing, successfully inhibited noxious heat avoidance and chemical avoidance of irritants, such as allyl isothiocyanate (AITC), a TRPA1 agonist commonly found in mustard oil (Arenas et al., 2017). Heat avoidance experiments consisted of opposing floor tiles of two different temperatures: noxious 32°C tiles that planarians mostly avoid and 24°C tiles that planarians mostly glide on. Inhibiting the expression of TRPA1 made planarians spend as much time on hot tiles as on room-temperature tiles. Planarians thus seem to detect noxious heat through TRPA1 but, in fact, further experiments suggested that heat does not directly activate TRPA1’s opening. The suggested cellular mechanism is rather an induction of reactive oxygen species (ROS) production that in turn can activate the TRPA1 ion channel (Arenas et al., 2017). This idea also corroborates with experiments on extraocular UV-light expositions in planarians, which induced noxious behavioral responses (by means of cellular ROS production) that were reduced in TRPA1-knockdown animals (Birkholz and Beane, 2017). Other examples of TRP knockdowns included targeting of planarian TRPM8 that resulted in a defect in thermotaxis for a wide range of cold temperatures (Inoue et al., 2014). Noxious cold avoidance experiments were done in similar conditions as for heat avoidance, but instead of tiles of different temperatures, a gradient was used from the side (non-noxious 25°C) to the center (noxious 17°C to 0°C) of a circular arena. Similarly, inhibition of the expression of TRPM8 induced more time spent in noxious cold areas than for wild-type animals. Moreover, genetic targeting of planarian TRPV1 reduced the noxious responses to the TRPV1 agonist and endocannabinoid anandamide. It did not, however, reduce capsaicin-induced responses, another well-known TRPV1 agonist, suggesting other receptors may be involved (Sabry et al., 2019).

### Modulation by opioids and other antinociceptive compounds

Lately, many antinociceptive compounds have been shown to modulate planarians’ responses in a similar way as in other animals. The first observation of opioids’ (i.e., morphine) effects on planarians behavior dates back to 1967 and has shown both tolerance and dependence characteristics (Needleman, 1967). By measuring the time an individual worm took to travel from the center of an illuminated petri dish to the side, Needleman demonstrated that when exposed to a high acute dose of morphine, the worm increased its transit time because of slow gliding or erratic movements. He then showed that this *hypokinesia* phenomenon could be gradually reduced to normal travel time after continuous exposure to morphine and that it could appear again after a withdrawal from chronic exposure (showing tolerance and dependence characteristic behaviors). Since then, other opioid agonists have been tested on planarians (e.g., DAMGO, a strong selective Mu opioid receptor agonist) with similar results and reversible effects using opioid antagonists (e.g., Naltrexone, a strong selective Mu opioid receptor antagonist) (Dziedowiec et al., 2018). However, to our knowledge, equivalents to opioid receptors still have not been specifically identified in planarians. In contrast, the presence of endogenous opioids (i.e., met-enkephalin) has been shown (Venturini et al., 1983). Many other synthetic opioid agonists and antagonists, as well as pro- and antinociceptive drugs have also been used in planarians. Examples include non-steroidal anti-inflammatory drugs such as meloxicam (Kim and Rawls, 2022), the polypeptide nociceptin (Rawls et al., 2008a), cannabinoid agonists or many other drugs of abuse mainly acting on monoamine and/or glutamatergic neurotransmission (Supplementary Table 1).

For further insights into behavior-modulating drugs in planarians, we listed in Supplementary Table 1 references that used behavioral tests or behavioral metrics in planarians and the stimuli they used. Supplementary Table 1 includes all references listed in section “Evidence for nociception in planarians” and “Planarian behavioral metrics” that modulated planarians’ behavior *via* a wide range of stimuli: chemical, mechanical, electric, thermic, or light. All chemicals are sorted based on the main known effect they have on nervous systems. Supplementary Table 1 is not expected to be an exhaustive list of the literature but rather a big entry point and a display of the wide variety of chemicals and stimuli used to modulate planarian behavior.

## Planarian behavioral metrics

Looking back into behavior, we noticed that the methods used in the literature to measure planarian locomotion can fall into two types of metrics: the quantitative and the qualitative metrics.

The *quantitative metrics* simply compared the distance traveled by planarians in a given time, either by counting the crossing of squares on a grid (Raffa et al., 2000, 2001, 2003, 2008, 2013; Raffa and Valdez, 2001; Rawls et al., 2006, 2007a,b, 2008a,b,c) or by measuring the total distance traveled by video tracking (Nishimura et al., 2007, 2011). The first one is usually noted as planarian LocoMotor Velocity (pLMV) or planarian LocoMotor Activity (pLMA).

The *qualitative metrics* are the observations of the body shape and the way planarians move. It is usually done either by identifying the main locomotion type (Carolei et al., 1975; Venturini et al., 1989), by grading the amount and intensity of multiple observed behaviors (Passarelli et al., 1999; Buttarelli et al., 2000) or by counting the occurrences of abnormal behaviors in a set time (Rawls et al., 2009, 2010, 2011; Pagán et al., 2012, 2015; Tallarida et al., 2014; Kim and Rawls, 2022). The latter was sometimes named planarian Seizure-Like Activity (pSLA) or planarian Seizure-Like Movements (pSLM) or simply “activity counts.”

Quantitative metrics, such as pLMV, had been introduced to propose and develop an objective measurement, independent of any experimenter bias. Indeed, those that have tried and analyzed the movements of planarians may have realized that they often display movements that are hard to characterize, often betwixt and between two types of locomotion that we want to identify, thus creating possible bias in the subjective interpretation of the movement and body shapes. However, if planarians sustain a clear type of locomotion for long enough, then the interpretation biases become negligible and the reaction to different compounds or concentrations can be compared more easily. The goal is to select the relevant properties of their movements to distinguish every possible form of reaction to heat, cold, irritants, narcotics, mechanical stimuli, etc. The best outcome would be to precisely characterize each movement with different criteria so that errors would be limited. At present time, only a few papers have tried to implement such classification of locomotion, and none of them seem to be used as a common baseline for further studies, even if some behaviors are commonly identified and used.

A great portion of the research using behavioral locomotion of planarians is presented in Table 1, where similar movement descriptions were associated to their respective designations by the authors. Table 1 shows that a few terms were used multiple times by independent research teams, such as “C-like position” or “Screw-like hyperkinesia.” Unfortunately, to the best of our knowledge, none of them properly defined these movements: they are mere descriptive terms of what the movement of the animal looked like. It is probable that different interpretations of the movement were made, such as for the term “headswing” which was used for two different gaits. All other terms mostly appeared only once. Some studies have implemented completely new descriptions of movements, but unfortunately none of them reached a consensual use in the field.

**TABLE 1:** Nomenclature of planarian noxious behaviors from the literature.

	Bardeen, 1901; Pearl, 1903	Corning and Freed, 1968	Welsh and Williams, 1970	Carolei et al., 1975; Palladini et al., 1981	Algeri et al., 1983	Venturini et al., 1989	Kimmel and Carlyon, 1990	Palladini et al., 1996; Passarelli et al., 1999	Buttarelli et al., 2000	Raffa and Desai, 2005	Nishimura et al., 2007	Farrell et al., 2008	Rawls et al., 2009, 2010; Tallarida et al., 2014; Kim and Rawls, 2022	Nishimura et al., 2011	Inoue et al., 2014	Cochet-Escartin et al., 2015; Arenas et al., 2017; Sabry et al., 2019
Normal locomotion	Swimming		Normal ciliary gliding motion	Normal	Normal	Normal	Normal gliding	Normal	Normal, slow movements	Normal	Smooth sliding and migration			Swimming	Normal	Gliding
Oscillation of body length, inchworm or caterpillar-like movement	Crawling		Inching				Looping			Clinging	Twisting and looping			Inching		Scrunching
Curling into a C-like shape		Sharp U turn		Plane curling		C-like tonic curling		C-like position	C-like position	Corkscrew	C-like hyperkinesia	C-like position	C-shapes, C-like hyperkinesia or hyperactivity			
Twisting along longitudinal axis				Screw-like hyperkinesia		Screw-like hyperkinesia		Screw-like hyperkinesia	Screw-like hyperkinesia	HeadSwing	Screw-like hyperkinesia	Screw-like hyperkinesia				
General augmentation of motor activities					Hyperkinesia										Hyperkinesia Lethargic	
Head and tail are anchored but mid-part is elevated				Tonic bending*					Bridge-like position							
Complete contraction of the body		Contraction							Walnut position						Crimpled	
Snake or S-like movements								Snake-like movement								
Tail is not anchored and twists										TailTwist						
Head is not anchored and nods		Head waving								HeadBop		Headswing				
Erratic, jerky, uncoordinated movements										Squirming			Twitching			
Stationary back and forth movements												Writhing				

References were chosen and associated based on the description of similar behavior the listed authors used. *The term “tonic bending” replaces the misspelled term “tonic bending” originally used by the authors (Carolei et al., 1975). Two references were not included in this table: (Hyman, 1951; Trueman, 1975). They supposedly included the term “looping” and were cited, respectively, by Kimmel and Carlyon (1990) and Cochet-Escartin et al. (2015).

**FIGURE 2:**
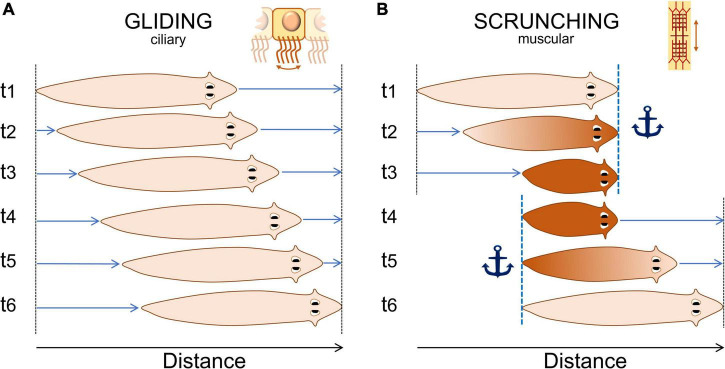
Normal “gliding” and noxious “scrunching” behavior representation. **(A)** Typical gliding behavior of planarians. They produce mucus and glide at a constant rate on a surface using ciliated cells located on their ventral side. Gliding speed is usually about 1–5 mm/s. **(B)** Typical noxious “scrunching” behavior. Scrunching is a muscular gait. The head is first anchored to the surface (t1), the body contracts (t2), anchor is switched from the head to the tail (t3 to t4), then the body elongates forward (t5), and loops to the first step (t6). The frequency of one loop varies between 0.2 and 0.8 Hz depending mostly on the species (Cochet-Escartin et al., 2015). Scrunching sometimes happen in reverse order, making the worm scrunch backward for a few seconds only.

The latest description of the “crawling” movement firstly observed more than a century ago and now referred to as “scrunching,” is so far the most detailed description of planarian noxious behavior (Cochet-Escartin et al., 2015). Scrunching is an almost-ubiquitous muscular type of gait induced by noxious stimuli. It is thought to be a defense mechanism triggered when normal gliding is impaired, such as from lesions, with distinct body movement characteristics (Figure 2). Authors have not only described the movements of this “scrunching” behavior but also characterized, for different species, the frequency, velocity and inducers of this behavior, among others. This study also proposed a disambiguation from another type of gait present in worm species: peristalsis. Peristalsis is another form of muscular locomotion that arises from a disruption of the planarian ciliated cells. It can be recognized by a short contraction of the body from the head to the tail. As the planarian moves forward, it looks as if the contraction bulge stays immobile relative to the substrate (Rompolas et al., 2010; Tanaka et al., 2012). Hence, we would suggest using the terms “gliding,” “scrunching,” and “peristalsis” in further studies of such behaviors. Concerning other types of locomotion in response to noxious stimuli, precise descriptions and disambiguations are awaited.

## Discussion and perspectives

While many arguments can already be found for planarian nociception, it should be noted that further experiments could be pursued to fully defend this concept.

To further describe nociceptive pathways in *planaria*, sensory neurons could be precisely traced. Fluorescent dye tracing of neural projections has already been done: it demonstrated, for example, that chemosensory neurons from the lateral branches project to the peripheral part of the brain (Okamoto et al., 2005; Agata, 2008). Precise tracing of neurons of interest for nociception, such as those that highly express TRPA1 or TRPV1 for example, has not been done yet, in part due to the lack of antibodies against planarian homologs of TRP channels. However, cells that express TRP channels genes can be marked by whole-mount *in situ* hybridization (Inoue et al., 2014). This technique allows precise mapping of cells that express specific families of TRP channels. One more step in nervous cells tracing is to be able to do it *in vivo* to facilitate electrophysiology recordings, which seems to be a hard task to accomplish (Koopowitz et al., 1996). We believe that nociceptive pathways could be traced using one or a combination of these techniques to further describe planarian sensory neurons.

With regards to electrophysiological properties of planarian nerve cells, most studies were performed by the Koopowitz team which used the polyclad flatworms *Freemania litoricola* (Koopowitz, 1975a,b) and *Notoplana acticola* (Bernardo et al., 1977; Keenan et al., 1979; Koopowitz et al., 1979a,b). These species are marine planarians, uncommon models that were solely used by this team. They performed extracellular recordings of both spontaneous and evoked activity mostly around the ventral nerve cords, from which results were discussed previously in section “Planarian research throughout centuries” (Sarnat and Netsky, 1985, 2002). More recently, one team has performed EEG-like recordings of the planarian brain (an electrode was inserted in the cerebral ganglions) which showed a low-frequency spontaneous activity, as well as short and loosely coupled neural feedback loops in *D. japonica* (Aoki et al., 2009). Hence, while new data is very scarce, it is still promising as it already replicated some of the older experimental results using common freshwater planarian models. The model could benefit from new replications of electrophysiological recordings, both to confirm the data in common lab planarians, and to make the most of the current technology and advances in the field. For example, simple patch-clamp recordings of single sensory neurons have not been done in planarians to the best of our knowledge. These recordings could eventually lead to the description of different functional families of sensory neurons, similar to those of other invertebrates that have been described. This would be a necessary step to confirm the physiological properties of nociceptors in planarian sensory neurons, such as sensitization, slow adaptation, high threshold, etc.

Moreover, multiple types of stimuli could be used to precisely decipher their nociceptive mechanisms. Indeed, *planaria* is especially convenient for chemically induced nociceptive behavior testing (Sabry et al., 2019), but nociceptive behavior has shown to be induced by many types of stimuli. They include amputation or electrical shocks (Cochet-Escartin et al., 2015), UV light (Birkholz and Beane, 2017) and noxious heat (Arenas et al., 2017) or cold (Inoue et al., 2014). Hence, electrophysiological recordings and screening of antinociceptive drugs could be adapted to multiple stimuli of interest (i.e., mechanical, thermal, inflammatory, etc.).

The high practicability of the *planaria* model led us, and others, to believe that planarians could be used in screening fields that need a high number of animals, such as environmental toxicology (Wu and Li, 2018) or high throughput screenings (Ireland et al., 2020). Toxicology studies have also previously showed that the lethality of dozens of different chemicals on hundreds of animals could be tested in parallel (Li, 2013). Later studies showed that not only lethality but also regeneration capacities and behavior (such as velocity or thermotaxis) could be analyzed in large quantities for neurotoxicology screening (Hagstrom et al., 2015). As previously discussed, the *planaria* model is also particularly adapted to poly-drug applications (Raffa, 2008). These ideas could lead to further large quantity screenings of compounds from chemotheques waiting for first-line *in vivo* testing, such as new analgesics, anti-inflammatory drugs, metabolites or other synthetic compounds.

We should note that, even if *planaria* is a particularly useful model for high throughput screening, there is a need for replication of results in the field. As we discussed, the term *planaria* represents at least 3 to 4 common species used in research and even more including less common ones. Japanese research teams would work mostly on *D. japonica*, American teams on *G. dorotocephala* or *G. tigrina* and European teams on *S. mediterranea*, for example. However, there is evidence that species react differently to some stimuli. *G. dorotocephala* seemed to be less photophobic than *G. tigrina* (Debold et al., 1965; Reynierse, 1967). *D. japonica* had higher locomotion velocities and frequency of “scrunching” behavior following any type of stimuli than *S. mediterranea* (Cochet-Escartin et al., 2015). *D. japonica* showed less lethality and more motility than *S. mediterranea* and *G. tigrina* after extensive storage, making them particularly adapted to high-throughput screening (Ireland et al., 2020). The need for standardization is obvious, but when one specie is chosen over the others for practical or scientific purposes, results should be interpreted carefully and conclusions should emphasize the specificities of the model.

If we want to look back at other nociceptive receptors, there is little to no data available. Of course, as we have seen in Supplementary Table 1, many pro- or antinociceptive compounds have been tested on planarians’ behavior. Beyond the scope of behavior, other compounds involved in the vertebrates’ nociception system have also been tested on planarians, such as substance P, which seemed to be a potent mitogen for planarian regeneration (Saló and Baguñà, 1986). However, still little is known about the presence of receptors of such compounds in planarians apart from TRPs. Examining available transcriptomes shows that ASIC4 and PIEZO receptors, respectively, acid-sensing and mechano-sensitive receptors, may be present, indicated by *in silico* gene prediction in multiple *S. mediterranea* transcriptomes (Rozanski et al., 2019). Homologies for P2X channels, P substance receptor NK1R or even opioid receptors are yet, on the other hand, unknown.

Ultimately, it has to be reported that a majority of experiments discussed in this review exposed planarians only to single condition, or to only single stimulus (one chemical, one wavelength, one electric shock, etc.). In the context of nociception, it would be relevant to apply multiple stimuli. Indeed, as we discussed, nociceptive systems usually show great plasticity, which was partially confirmed by electrophysiology recordings in marine planarian models. Adaptation, sensitization and desensitization are such examples of responses that need multiple sets of tests to appear and that are relevant in a drug screening context, especially for chronic drug exposure.

To briefly conclude, we would stress that, beyond the field of regeneration, planarians constitute a promising model to study the molecular mechanisms behind nociception in invertebrates and beyond.

## Author contributions

GR performed the literature search. GR and HC wrote the manuscript. HC designed the figures. VL contributed to the review process of the manuscript and handed out valuable feedback and relevant revisions. All authors contributed to the article and approved the submitted version.

## Supplementary material

Supplementary Table 1 List of the chemicals or stimuli used to modulate planarians’ behavior from references discussed in this review. Chemicals are sorted based on their known main action on nervous systems. Attached is a list of succinct description of each chemical from the table to help apprehend their role in its corresponding study.

## Abbreviations

TRPs: Transient Receptor Potential ion channels
*G*.: *Girardia*
*D*.: *Dugesia*
*S*.: *Schmidtea*
ROS: Reactive Oxygen Species
pLMV: planarian LocoMotor Velocity
pLMA: planarian LocoMotor Activity
pSLA: planarian Seizure-Like Activity
pSLM: planarian Seizure-Like Movements
AITC: Allyl IsoThioCyanate
dsRNA: double-stranded RNA
RNAi: interference RNA technology.
