# Supplementary material for: Planarian nociception: Lessons from a scrunching flatworm
Source: Front Mol Neurosci. 2022 Jul 26;15:935918. doi: 10.3389/fnmol.2022.935918 (PMC9362985; doi:10.3389/fnmol.2022.935918)
Supplement: Supplementary Table 1 — List of the chemicals or stimuli used to modulate planarians’ behavior from references discussed in this review. Chemicals are sorted based on their known main action on nervous systems. Attached is a list of succinct description of each chemical from the table to help apprehend their role in its corresponding study. [file Table_1.DOCX]

|  | Act on TRPs | Adrenergic | Glutamatergic | Dopaminergic | Serotonergic | Cholinergic | GABAergic | Opioidergic | Others or undefined targets |
| --- | --- | --- | --- | --- | --- | --- | --- | --- | --- |
| **Bardeen 1901** |  |  |  |  |  |  |  |  | Touch (cut/poke) |
| **Pearl 1903** |  |  |  |  |  |  |  |  | Strong chemicals (undefined), Touch, Electric currents |
| **Corning 1968** |  | Reserpine | Reserpine | Reserpine | Reserpine |  |  |  |  |
| **Carolei 1975** |  | Reserpine, Clonidine | Reserpine | Reserpine, L-Dopa, Benserazide, Benserazide, Piribedil, Apomorphine, Haloperidol | Reserpine |  |  |  |  |
| **Palladini 1981** |  | Reserpine | Reserpine | Reserpine, Haloperiod | Reserpine |  |  |  | Eosine, Hematoporphyrin |
| **Algeri 1989** |  | Reserpine | Reserpine | Reserpine, Haloperidol, Apomorphine, L-Dopa | Reserpine |  |  |  |  |
| **Venturini 1989** |  | Reserpine, Amphetamine | Reserpine, Amphetamine | Reserpine, CY 208-243, SKF 38393, PHNO, LY 141865, Lisuride, Terguride, Apomorphine, Amphetamine, SCH 23390, Sulpiride, Haloperidol | Reserpine, Amphetamine |  |  |  |  |
| **Kimmel 1990** |  |  |  |  | pCPA |  |  |  |  |
| **Palladini 1996** |  | Reserpine, Nomifensine | Reserpine | Reserpine, Cocaine, Nomifensine, Sulpiride, SCH 23388, SKF 38393 | Reserpine |  |  |  |  |
| **Passarelli 1999** |  |  |  | Sulpiride, SCH-23390 |  |  |  | U50,488, Bremazocine, DAMGO, DPDPE, Naloxone, Nor-BNI |  |
| **Buttarelli 2000** |  |  |  | Apomorphine, Nomifensine, SCH-23388, Sulpiride, Haloperidol |  | Physostigmine, Nicotine, Atropine |  |  |  |
| **Raffa 2000** |  |  |  | Sulpiride |  |  |  |  | Light : UV-L (366 nm), UV-S (254 nm) |
| **Raffa and Valdez 2001** |  |  |  | Cocaine |  |  |  |  | Light : UV-L (366 nm), UV-S (254 nm) |
| **Raffa 2001** |  |  |  | Sulpiride |  |  |  |  |  |
| **Raffa 2003** |  |  |  |  |  |  |  | U-50,488H, Naloxone, Nor-BNI |  |
| **Raffa 2005** |  |  |  | Cocaine |  |  |  |  |  |
| **Rawls 2006** |  |  |  | Cocaine |  |  |  |  | WIN 55212-2, L-NAME |
| **Nishimura 2007** |  | Methamphetamine | Methamphetamine | Dopamine, Bromocriptine, Methamphetamine | Methamphetamine |  |  |  |  |
| **Rawls 2007a** | Icilin, Capsaicin |  |  |  |  |  |  |  |  |
| **Rawls 2007b** |  |  |  | LY 235959 |  |  |  |  | WIN 55212-2 |
| **Farrell 2008** |  |  |  |  | 8-OH-DPAT, mCPP, 5-HT, WAY-100635 |  |  |  |  |
| **Raffa 2008** |  | Amphetamine | Amphetamine | Amphetamine | Amphetamine |  |  | Naloxone, Nor-BNI, CTAP, Naltrindole | WIN 52212-2 |
| **Rawls 2008a** |  | Methamphetamine | Methamphetamine | Methamphetamine | Methamphetamine |  |  | JTC-801 | Nociceptin |
| **Rawls 2008b** |  | Amphetamine, Methamphetamine | Amphetamine, Methamphetamine, Ceftriaxone | Amphetamine, Methamphetamine, Cocaine | Amphetamine, Methamphetamine |  | Clorazepate |  | Ceftriaxone |
| **Rawls 2008c** |  |  |  |  |  |  |  | U-50,488 | Agmatine, WIN 55,212-2, Arginine |
| **Rawls 2009a** |  | Propranolol | DNQX, Topiramate, AMPA, Glycine, NMDA, MK-801 |  |  |  |  |  |  |
| **Rawls 2009b** |  |  | NMDA, MK-801, DNQX, Topiramate, AMPA |  |  |  |  |  |  |
| **Rawls 2010** |  |  | Glutamate | Cocaine |  | Scopolamine | GABA |  | Caffeine |
| **Rawls 2011** |  |  |  |  |  | Nicotine |  |  |  |
| **Nishimura 2011** |  |  |  |  |  |  |  |  | Ethanol |
| **Pagan 2012** |  | Amphetamine | Amphetamine, Glutamate, NMDA | Cocaine, Amphetamine | Amphetamine, Parthenolide | Nicotine, Cytisine |  |  | Parthenolide |
| **Raffa 2013** |  |  |  |  |  | Nicotine, Mecamylamine, Scopolamine |  | Naloxone, CTAP, Naltrindole, nor-BNI |  |
| **Inoue 2014** |  |  |  |  |  |  |  |  | Temperature ranges from 0°C to 35°C |
| **Tallarida 2014** |  |  |  | Cocaine |  | Nicotine |  |  | Ethanol |
| **Cochet-Escartin 2015** |  |  |  |  |  |  |  |  | Ficoll, Low pH, Touch (cut), High temperature (32°C), Electric shocks |
| **Pagan 2015** |  |  |  |  |  | Nicotine, Curare |  |  |  |
| **Dziedowiec 2018** |  |  |  |  |  |  |  | DAMGO, U50,488, Naltrexone, Nor-BNI, Morphine |  |
| **Sabry 2019** | AITC, Capsaicin, Anandamide, HC-030031, SB-366791 |  |  |  |  |  |  |  | H2O2, HCl |
| **Zewde 2018** |  | Mephedrone | Mephedrone | Mephedrone, Bupropion | Mephedrone, Bupropion, Fluoxetine |  | Clorazepate, FG-7142 |  | Ethanol |
| **Kim 2022** | HC-030031 |  |  |  |  | Nicotine, Pancuronium |  | DAMGO, Naloxone | Meloxicam |

**5-HT :** serotonin, neurotransmitter

**8-OH-DPAT hydrobromide :** 5-HT receptor agonist

**Agmatine :** resembling arginine

**AITC (allyl isothiocyanate) :** TRPA1 agonist

**AMPA :** AMPA-receptor agonist (mimics glutamate)

**Anandamide :** TRPV1 agonist

**Apomorphine :** D2 agonist

**Atropine :** muscarinic acetylcholine receptors antagonist

**(Met-)Amphetamine :** mainly monoamine releasing agents

**Benserazide :** dopa decarboxylase inhibitor

**Bromocriptine :** mainly a partial D2 agonist

**Bremazocine :** Kappa Opioid Receptor (KOR) agonist

**Bupropion :** serotonin and dopamine reuptake inhibitor

**Caffeine :** adenosine receptors antagonist

**Capsaicin :** TRPV1 agonist

**Ceftriaxone :** beta-lactam antibiotic

**Clonidine :** alpha2-adrenergic agonist

**Clorazepate :** benzodiazepine (enhances the action of GABA)

**Cocaine :** mainly blocks the dopamine reuptake transporters

**CTAP :** Delta Opioid Receptor (DOR) antagonist

**Curare :** nicotinic acetylcholine receptor antagonist

**CY 208,243 :** D1 agonist

**Cytisine :** nicotinic partial agonist

**DA :** dopamine, neurotransmitter

**DAMGO :** Mu Opioid Receptor (MOR) agonist

**DNQX :** AMPA-receptor antagonist

**DPDPE :** DOR agonist

**Eosin :** photosensitizer

**Ethanol :** alcohol

**FG-7142 :** benzodiazepine

**Ficoll :** polysaccharide

**Fluoxetine :** selective serotonin reuptake inhibitor (sSRI)

**GABA :** inhibitory neurotransmitter

**Glutamate :** amino acid, neurotransmitter

**Glycine :** amino acid, neurotransmitter

**H2O2 :** Reactive Oxygen Species (ROS) compound

**Haloperidol :** D2 antagonist

**HC-030031 :** TRPA1 antagonist

**HCl :** acid

**Hematoporphyrin :** photosensitizer

**Icilin :** TRPM8 agonist

**JTC-801 :** nociceptin receptor (NOP / ORL-1) antagonist

**L-Dopa :** dopamine precursor

**Lisuride :** D2 selective agonist

**L-NAME :** nitric oxide synthesis inhibitor

**LY 141865 :** D2 selective agonist

**LY 235959 :** NMDA antagonist

**mCPP :** 5-HT agonist

**Mecamylamine :** nicotinic acetylcholine receptors antagonist

**Meloxicam :** NSAID

**(S-)Mephedrone :** monoamine releasing agent, similar to amphetamines

**MK-801 :** NMDA-receptor antagonist

**Morphine :** opioid receptors agonist

**Naloxone :** opioid receptors antagonist

**Naltrexone :** MOR antagonist

**Naltrindole :** DOR antagonist

**Nicotine :** nicotinic acetylcholine receptors agonist

**NMDA :** amino acid, glutamate-mimicking

**Nociceptin :** nociceptin receptor (NOP / ORL-1) agonist

**Nomifensine :** norepinephrine-dopamine reuptake inhibitor

**Nor-BNI :** selective KOR antagonist

**Pancuronium :** non-depolarizing curare

**Parthenolide :** multiple targets, anti-inflammatory

**pCPA (fenclonine) :** inhibitor of tryptophan hydroxylase (serotonin depletor effect)

**PHNO :** D2 selective agonist

**Physostigmine :** cholinesterase inhibitor

**Piribedil :** D2 and D3 agonist

**Propranolol :** beta-adrenergic antagonist

**Reserpine :** mono-amine depleting agent

**SB-366791 :** TRPV1 antagonist

**SCH 23390** and **SCH 23388 :** D1 selective antagonists

**Scopolamine :** muscarinic antagonist of muscarinic acetylcholine receptors

**SKF 38393 :** D1 agonist

**Sulpiride :** D2 and D3 antagonist

**Terguride :** D2 agonist

**Topiramate :** inhibition of glutamate-activated ion channels in this context

**U-50,488 :** selective KOR agonist

**WAY-100635 maleate :** 5-HT1 antagonist

**WIN 52212-2 :** cannabinoid agonist
